# Multifocal chondrosarcoma of the hand: Case report and review of the literature

**DOI:** 10.1002/ccr3.4352

**Published:** 2021-06-09

**Authors:** Hunter B. Jones, Jacob Murphree, Joash R. Suryavanshi, Bradley O. Osemwengie, Sterling Rosqvist, Cameron T. Cox, Brendan J. MacKay

**Affiliations:** ^1^ Texas Tech University Health Sciences Center Lubbock TX USA; ^2^ University Medical Center Lubbock TX USA

**Keywords:** chondrosarcoma, hand malignancy, multifocal tumor, reconstructive hand surgery

## Abstract

Few multifocal hand chondrosarcomas have been reported. To our knowledge, this report is the first to describe multifocal hand chondrosarcoma in a patient with no evidence of prior enchondroma, Ollier's disease, or Maffucci syndrome.

## Graphical abstract

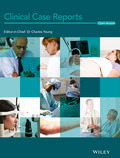

## 1 INTRODUCTION

Reported cases of multifocal hand chondrosarcoma have arisen from enchondromas in patients with Ollier's disease, Maffucci syndrome, or hereditary exostosis. We report a multifocal hand chondrosarcoma with no evidence of prior enchondroma. Malignant tissue was resected with wide margins, and there was no recurrence or metastasis at 28 months.

Chondrosarcomas are the third most common primary malignant bone tumors behind myeloma and osteosarcoma.^1^ These neoplasms are derived from cartilage and typically arise during the 5th or 6th decade of life, presenting as an enlarging mass with or without pain.^2^, ^3^ They are most often found in long bones such as the femur and humerus, as well as the pelvic bones.

Chondrosarcomas have been reported in the hand, but are seen much less frequently.^4^ In a series of 11,087 cases, Unni and Dahlin found that approximately 1.5% of chondrosarcomas involved the hand and wrist,^5^ and smaller studies have reported up to 8% in the literature.^6^

Primary bone tumors are rarely found in the wrist and hand.^2^ In a series of 7975 bone tumors, only 7% of benign tumors and 0.5% of malignant tumors presented in this region.^7^ Despite their rare occurrence relative to other locations, chondrosarcomas are the most frequently occurring bone malignancy of the hand, accounting for approximately 40% of all malignant bone tumors in this location.^1^, ^8^

En bloc surgical resection is the current gold standard of treatment for low‐, middle‐, and high‐grade chondrosarcomas.^9^, ^10^ These tumors are notoriously resistant to chemotherapy and radiation,^2^ possibly due to the increased expression of p‐glycoprotein and telomerases, as well as low rates of division and blood supply characteristics.^11^ Furthermore, local resection is usually ineffective, with recurrence rates reported as high as 92%. Thus, removing chondrosarcomas requires wide resection margins, and tumor resection coupled with amputation and radical resection has reduced the rate of recurrence to 16%.^10^, ^12^

Chondrosarcomas are most often singular and do not typically arise in a multifocal pattern. At times, chondrosarcomas of the hand occur secondary to conditions such as Ollier's disease, Maffucci syndrome, or hereditary exostosis.^13^ In these rare cases, preexisting enchondromas undergo malignant conversion to chondrosarcoma.

In a 352 patient series, Douis et al found that less than 1.6% (3 cases) of all chondrosarcomas demonstrated multifocal distribution.^14^ Furthermore, only two multifocal presentations of hand chondrosarcoma have been reported in the literature, both of which arose from preexisting enchondromas.^15^, ^16^ We present the case of a multifocal chondrosarcoma of the ulnar aspect of the left hand. Our patient did not have Ollier's disease, Maffucci syndrome, or hereditary exostosis and had no evidence of prior enchondroma. He was treated with tumor resection including a partial hand amputation and ray resection of the 5th digit.

## 2 METHODS

A 32‐year‐old man was referred to our clinic with slowly increasing pain and swelling over the PIP joint of the 5th digit in his left hand. The patient stated that he had jammed his finger in a basketball game 16 years ago, and the pain had gradually gotten worse to the point that he could no longer flex his small finger at the PIP joint.

**FIGURE 1:**
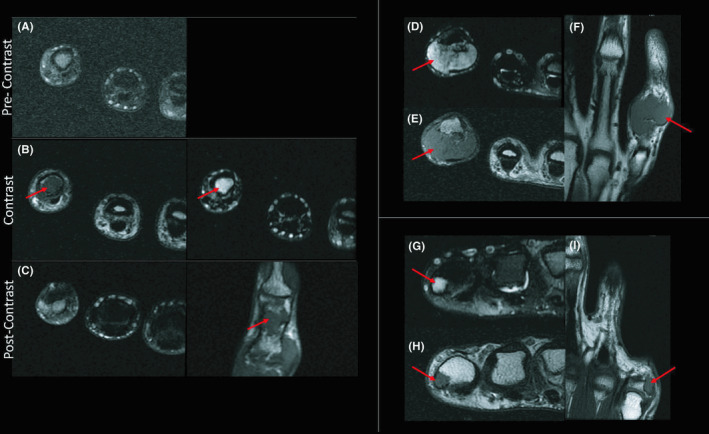
Original MRI showing an enhancing lesion with distinct areas of T2 hyperintensity and T1 hypointensity (B, C: arrows) separated by regions of T1 hyperintensity. MRI showing a T2 hyperintense and T1 hypointense (G‐I: arrows) lesion measuring 0.5 x 0.6 x 0.9 cm on the ulnar base of the 5th proximal phalanx

MRI at the time of presentation showed a 2.2 × 1.1 × 4.1 cm mass extending along the volar 5th digit from the mid proximal portion to the base of the distal phalanx between the bones and the fifth flexor tendon. This lesion had induced cortical thinning and scalloping in the distal half of the fifth middle phalanx. (Figure 1) Multiple areas of T1 hypointensity were thought to correspond to foci of mineralization within the soft tissue mass. MRIs also showed an enhancing lesion measuring 0.5 × 0.6 × 0.9 cm along the ulnar aspect of the base of the fifth proximal phalanx. (Figure 1g‐i).

**FIGURE 2:**
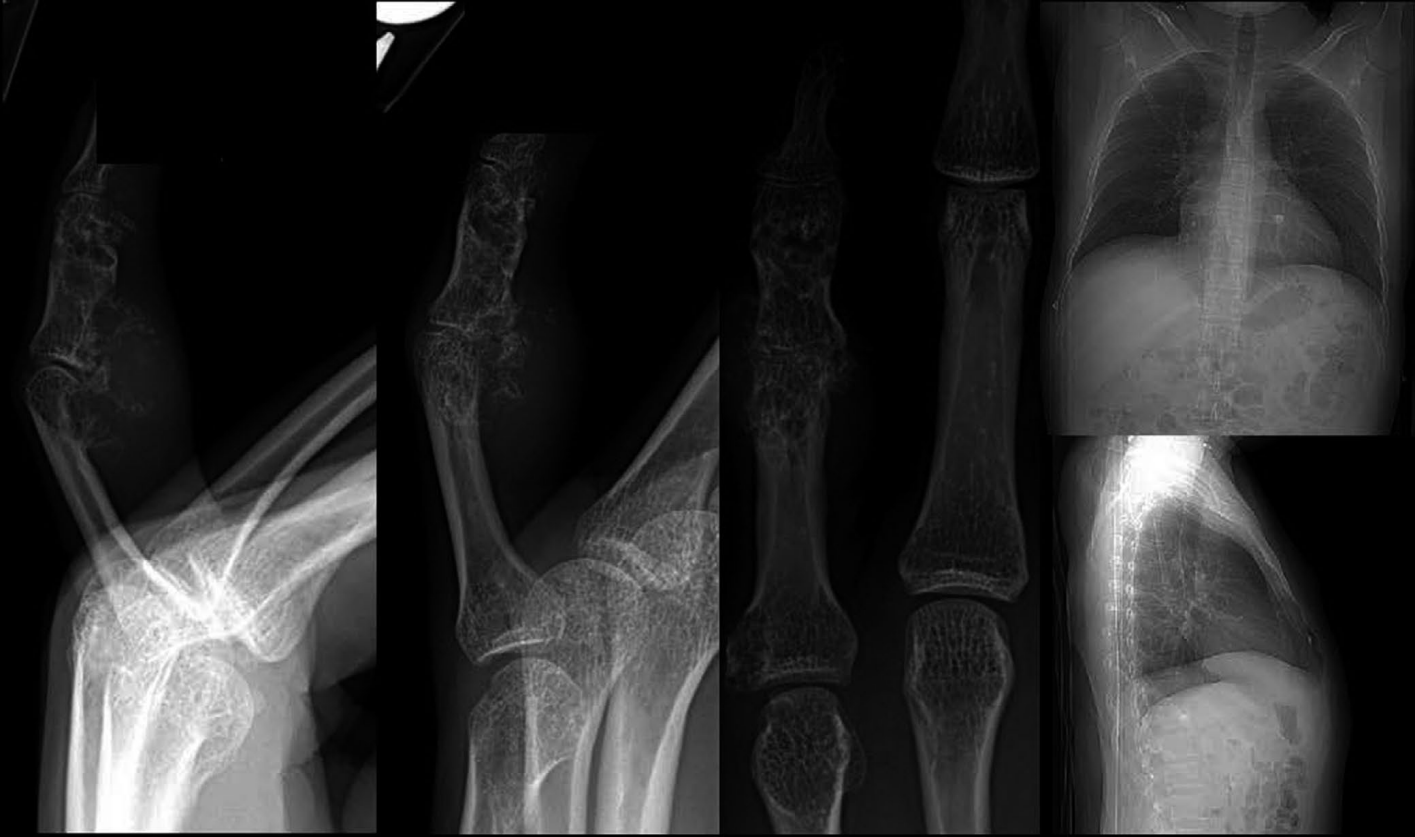
Preoperative radiographs showing abnormality in the small finger

**FIGURE 3:**
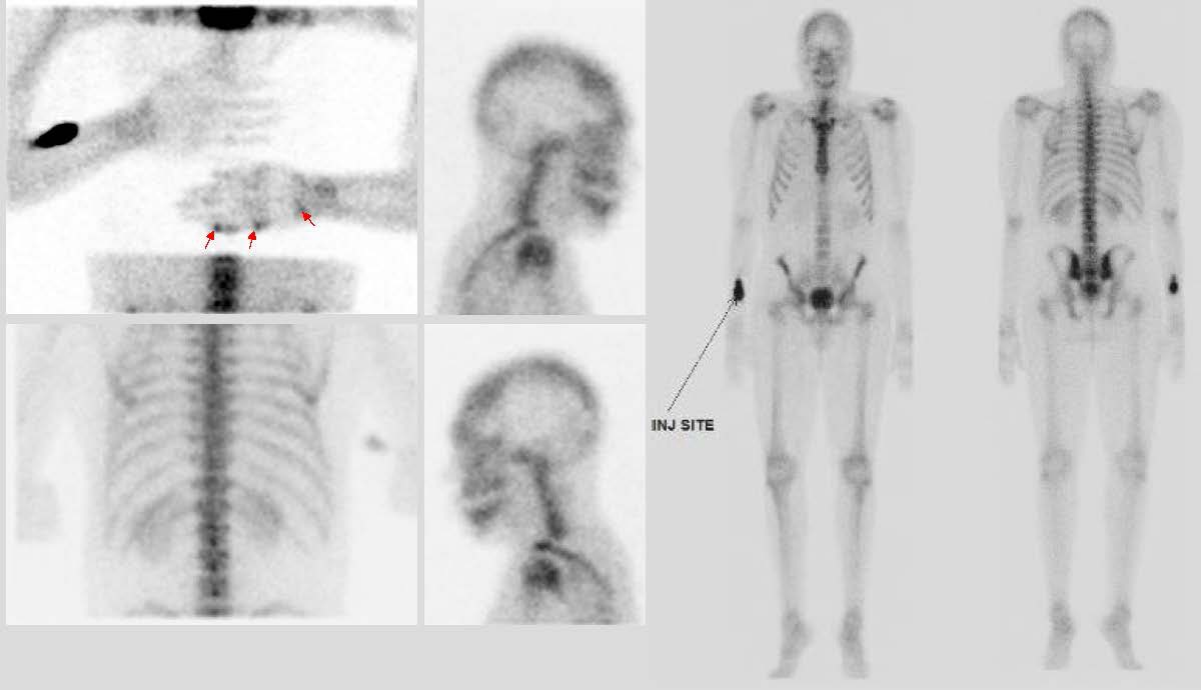
Bone scan showing focal areas (arrows) in the in the 5th digit and in the wrist

**FIGURE 4:**
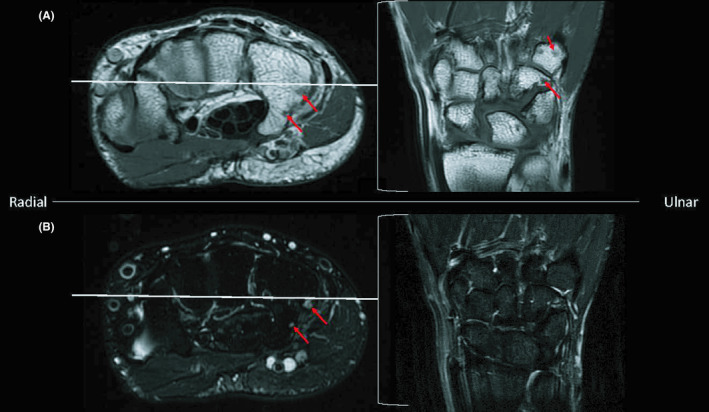
MRI showing T1 hypointense and T2 hyperintense (arrows) 6‐mm lesion in the hook of the hamate, as well as a 3‐mm body in the ulnar body of the hamate and a 4‐mm lesion in the ulnar base of the proximal metacarpal (arrows)

After initial evaluation, the decision was made to proceed with further imaging including: radiographs (Figure 2), bone scans (Figure 3), and MRI (Figure 4). Bone scans showed two focal areas in the 5th digit, as well as two focal areas in the wrist. (Figure 3).

Repeat MRI showed a lesion within the hook of the hamate measuring approximately 6 mm (Figure 4), as well as one punctate lesion in this area. An additional lesion was found within the ulnar body of the hamate measuring approximately 3 mm. (Figure 4). A small lesion in the proximal aspect of the 5th metacarpal measuring approximately 4 mm was found (Figure 4) with an additional punctate lesion in the area. These images also confirmed the presence of a lesion in the ulnar base of the fifth proximal phalanx, as seen on previous MRI.

A subsequent biopsy of the mass in the proximal phalanx and metacarpal identified these as low‐grade chondrosarcoma with features including: hypercellularity, chondrocytes with mild nuclear atypia, and binucleated chondrocytes.

After consultation with an oncologist‐hematologist, wide resection with clear margins was recommended as smaller margins may leave residual tumor, and these tumors are typically unresponsive to chemotherapy and radiation. The patient elected to undergo partial hand amputation which would include resection of the fifth ray and the affected portions of carpals and metacarpals, as well as a groin flap for closure.

Ray resection of the 5th digit was performed, and the proximal portion of the 4th metacarpal containing the tumor was removed. The hamate and an affected portion of the capitate were also removed, with all malignant tissue removed en bloc. The radiocarpal joint had become unstable secondary to resection and was stabilized with percutaneous pins. Two screws were placed from the residual 4th metacarpal to the 3rd metacarpal to provide stability. Next, the groin flap was lifted, and the hand was positioned and sewn into place. One month later, the groin flap was transected and inset.

**FIGURE 5:**
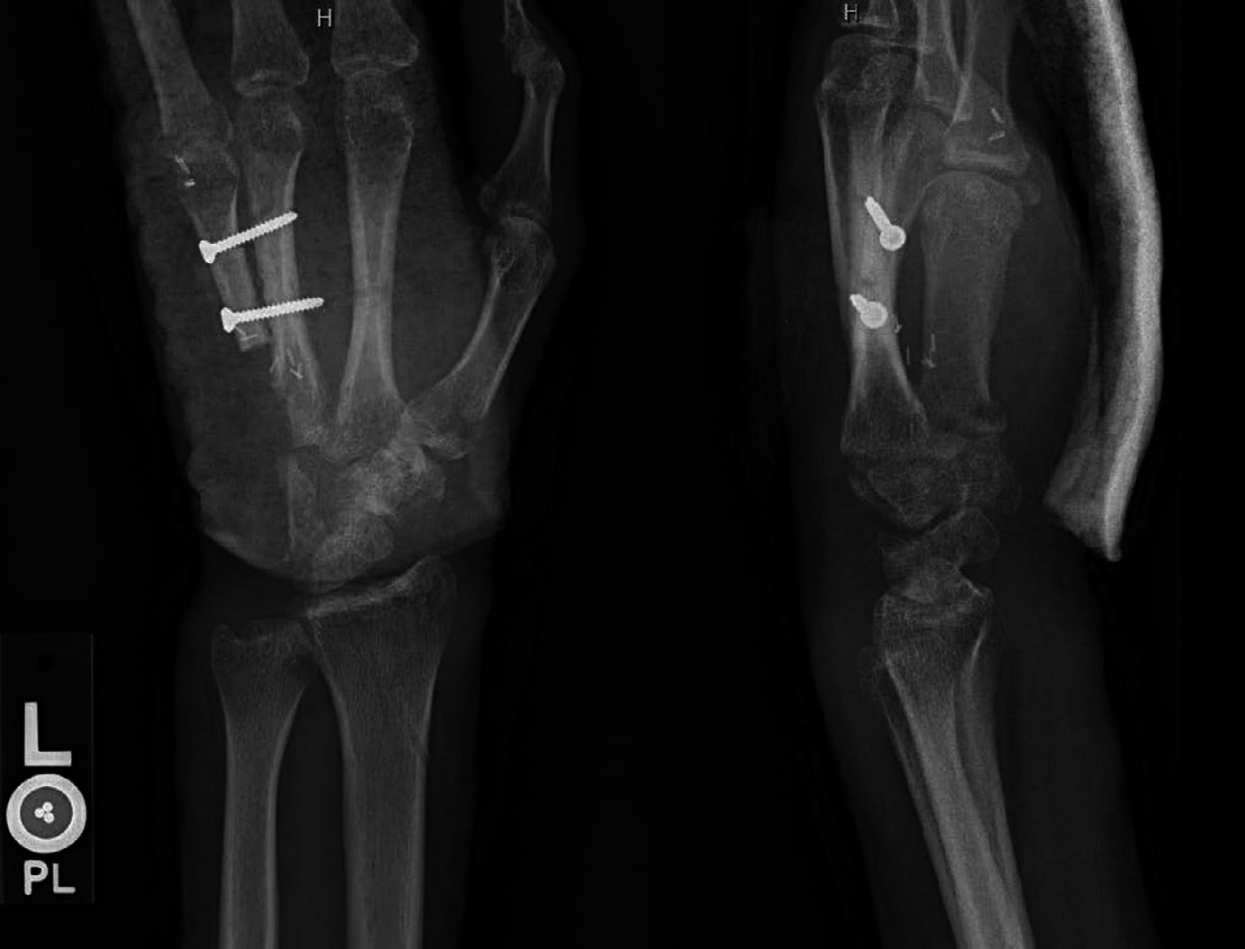
Intraoperative radiographs following removal of percutaneous pins and manipulation for improved range of motion

Two months after the initial surgery, the patient returned to the OR for removal of hardware. While the patient was under anesthesia, the wrist, index, and middle fingers were manipulated to reduce stiffness. After the pins were removed, final radiographs were taken (Figure 5). The patient was placed into an extension splint to prevent contracture.

**FIGURE 6:**
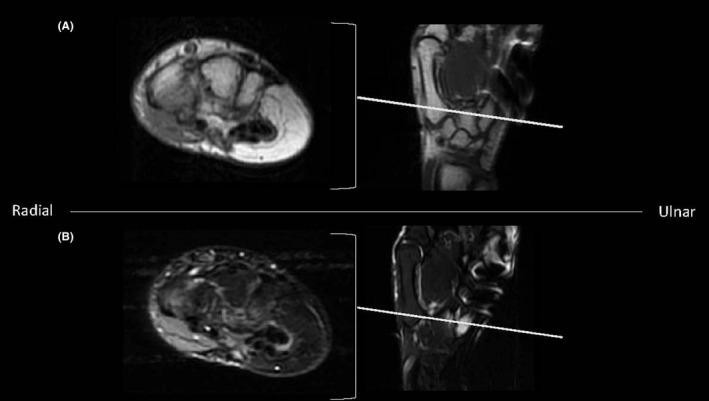
14‐month postoperative MRI (without contrast) showing (A) T1 and (B) T2 images of the left hand

Subsequently, he continued to have stiffness and functional deficits as he was unable to attend scheduled therapy visits for an extended period of time. At 14 months, MRI showed an absence of residual chondrosarcoma (Figure 6). Surgery was scheduled for MCP capsulectomy, rotational osteotomy, and tenolysis of the IF, LF, and RF flexor tendons to improve range of motion. Due to the patient's work constraints, however, this operation was canceled, and the patient was not seen for another 6 months.

**FIGURE 7:**
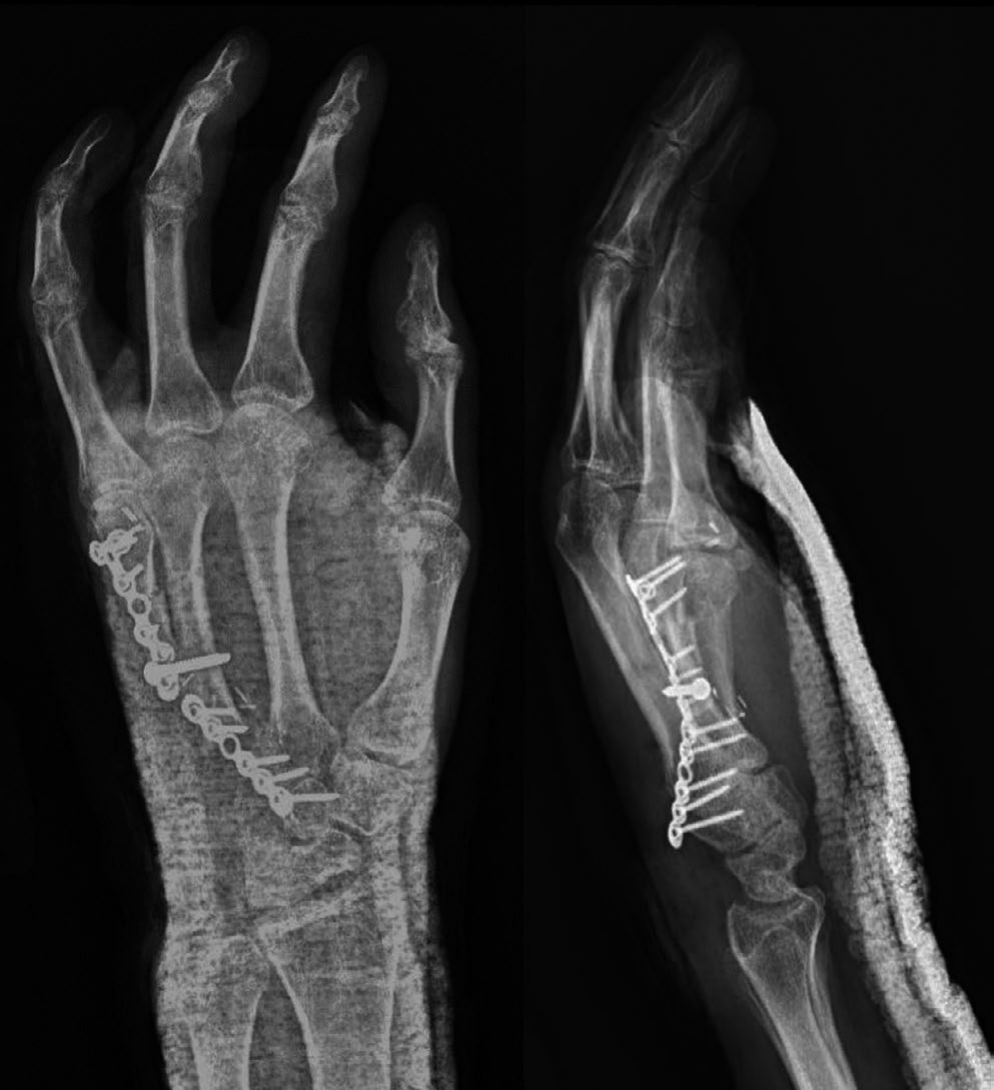
Intraoperative radiographs taken 20 months after the initial resection procedure

Twenty months after the initial resection and reconstruction, tenolysis, capsulectomy, and rotational osteotomy were completed. At this time, we did not find any evidence of recurrence or metastasis intraoperatively or on radiographs (Figure 7).

## 3 RESULTS

**FIGURE 8:**
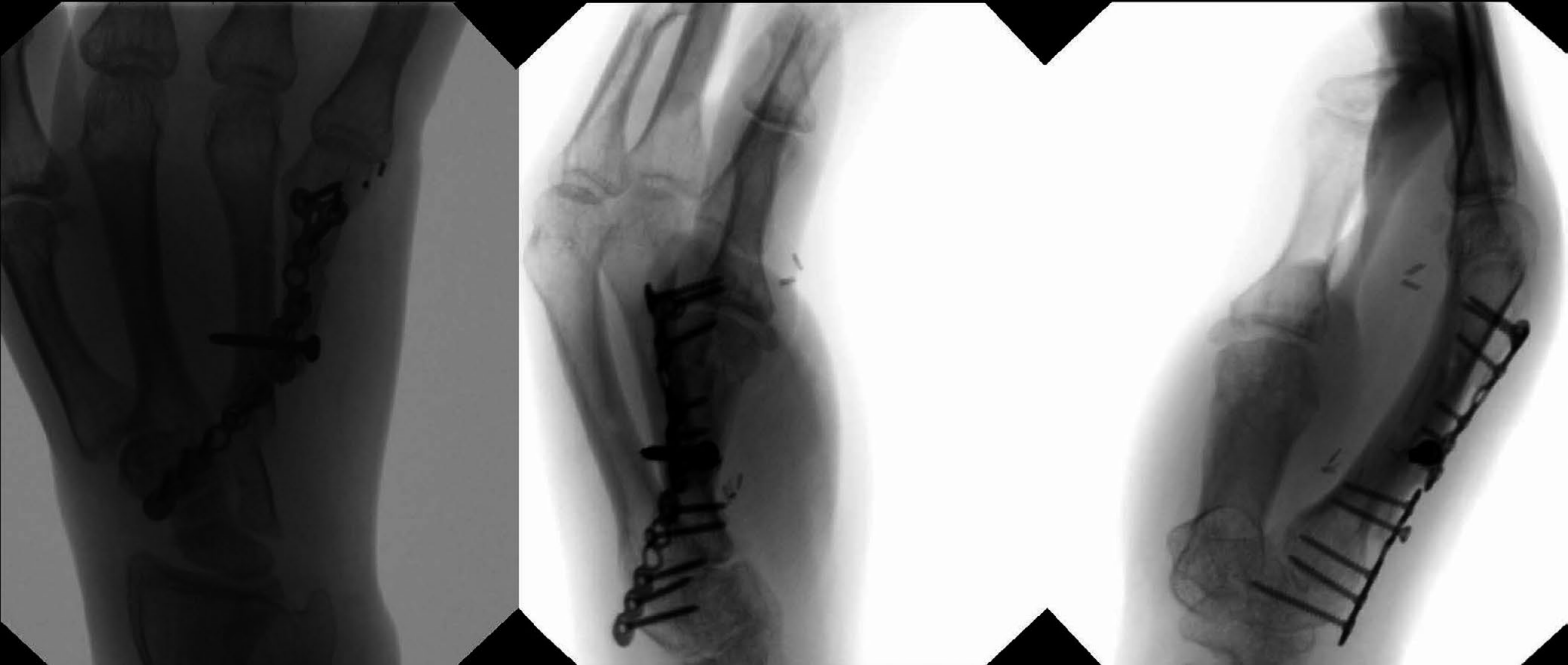
Fluoroscopic images taken 21 months after the initial resection procedure

Twenty‐one months after the initial operation, the patient was progressing as expected. He reported minimal pain and had returned to work without issue. At this visit, he lacked 60° range of motion at the MCP with 2.5‐cm pulp‐to‐palm deficiency, but was still improving. Fluoroscopic images confirmed that all hardware remained in place, and there was no evidence of recurrence and/or metastasis at this time (Figure 8).

Twenty‐six months post‐resection, the patient was seen in the urgent care clinic for an infected blunt laceration wound to his left hand sustained 1 month prior to this visit. He was given bactrim and scheduled to follow up in clinic.

**FIGURE 9:**
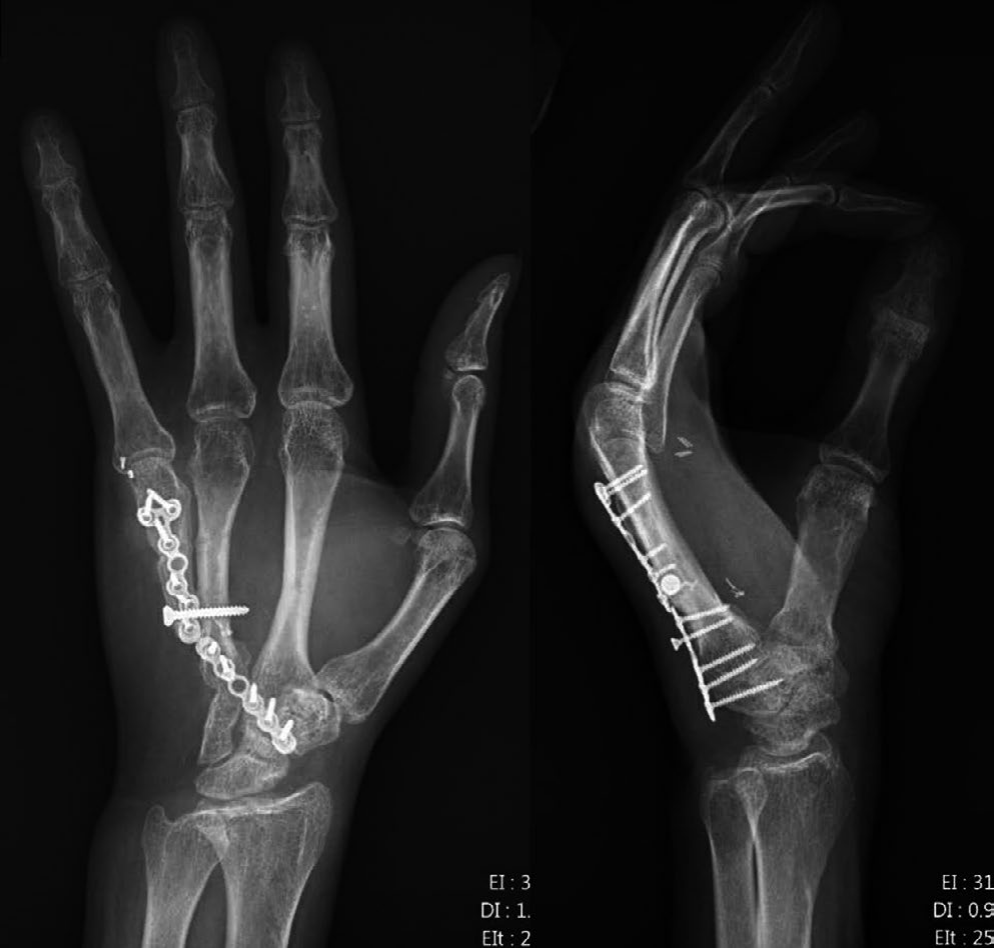
Radiographs taken 28 months after initial resection procedure

At 28 months, the patient returned to clinic for follow‐up regarding the aforementioned wound. Radiographs taken at this visit showed that the hardware was well aligned with healing of the osteotomy, and there was no evidence of recurrence or metastasis (Figure 9). Grip strength was 80 lbs in the right hand and 15 lbs in the left (operative side). Key pinch, tip pinch, and 3‐jaw pinch were all 17 lbs on the right and 6 lbs on the left. He had 5‐cm pulp‐to‐palm deficiency, with full active flexion of the thumb, 110° flexion of the index finger, and 90° flexion in the long and ring fingers. His pain was fully resolved at this time, and he had returned to 50% of normal activities. QuickDASH and SF‐20 forms were completed at this time—QuickDASH = 47.7%, SF‐20: Physical Function = 83.3, Role Functioning = 75.0, Social Functioning = 100, Mental Health = 80.0, Health Perceptions = 82.2, Pain = 40.0. Our patient continues to attend physical therapy to improve ROM.

## 4 DISCUSSIONS

Given their infrequent occurrence, multifocal chondrosarcomas continue to present both diagnostic and prognostic challenges. The histological criteria of chondrosarcoma and enchondroma are not easy to distinguish. This often leads to the misdiagnosis of chondrosarcoma as enchondroma.^17^ Chondrosarcoma lesions of the hand commonly show cortical destruction and bone expansion. Soft tissue extension that has begun to permeate bone and irregular cortical wall thickening are indications of malignant chondrosarcoma.^17^ In assessing cartilaginous legions of the hand, histology and radiology may both be necessary to correctly diagnose chondrosarcoma.

While local resection has been described in the literature, hand chondrosarcomas in these cases present with high rates of recurrence. ^10^, ^12^, ^18^ In studies where radical resection was favored, lower rates of recurrence were observed.^18^ This aligns with the historical understanding that choice of treatment influences rates of recurrence in these tumors. ^19^ For this reason, partial hand amputation was chosen over local resection in our case.

The metastatic potential chondrosarcomas in the hand are low compared to other locations.^8^, ^20^ Because of this phenomenon, some have suggested that they should be categorized separately from non‐hand chondrosarcomas.^21^ Low rates of metastasis have lead others to employ more conservative treatment algorithms to preserve function.^10^, ^22^ However, given their aggressive rate of local recurrence and resistance to chemotherapy and radiation, wide resection and regular postoperative screenings are still recommended.^2^, ^10^ While metastatic conversion is not impossible, it does not elicit any special consideration in treatment algorithms for hand chondrosarcomas.

At 26 months post‐resection, no such recurrence has been documented in our patient. The findings of our case align with several hand chondrosarcoma cases treated by ray resection and/or digital amputation, which found no evidence of recurrence 5‐34 months after the primary procedure.^23^, ^24^, ^25^, ^26^

Of three patients identified by Douis et al with multifocal chondrosarcoma, two had Ollier's disease.^14^ This condition, also known as enchondromatosis, is a genetic disorder associated with the development of numerus enchondromas in proximity to epiphyseal cartilage, mainly in the phalanges and metacarpals. This results in numerous well‐defined, disfiguring sclerotic lesions. It is possible for these enchondromas to undergo malignant transformation to chondrosarcomas.^27^ The third patient in the series by Douis et al, however, had no features to suggest Ollier's disease and developed synchronous multifocal chondrosarcomas on the pubis, proximal femur, and contralateral distal femur.^14^ Our patient did not show signs of prior enchondroma formation to suggest presence of Ollier's disease or Maffucci syndrome—another disease known to cause multifocal enchondromas which may then convert to chondrosarcoma.^10^, ^14^

We were able to find only two cases of multiple chondrosarcomas of the hand in the literature, both of which originated from enchondromas that underwent malignant conversion.^15^, ^16^ In our case, there was no evidence of prior enchondroma, Ollier's disease, or Maffucci syndrome. Some have suggested that chondrosarcomas of the hand often originate from preexisting enchondromas given their extended duration of symptoms and the fact that they present nearly three decades later in life than enchondromas.^18^ However, published data indicate that chondrosarcoma of the hand arising from enchondroma is extremely rare.^3^, ^10^

## 5 CONCLUSIONS

Due to the rare nature of this case, it is unlikely that a single institution will have a sufficient number to perform a study on this particular pattern of chondrosarcoma. As such, we recommend that institutions collaborate to form a case series investigating prognostic factors and treatment modalities for multifocal chondrosarcomas.

## CONFLICT OF INTEREST

Though they are not directly funding this case report, the authors would like to disclose the following support for BM: Paid teaching for TriMed. Paid teaching and consulting, as well as research support from AxoGen. Paid consulting for Baxter/Synovis and GLG. The remaining authors have nothing to disclose.

## AUTHOR CONTRIBUTIONS

HBJ: served as a primary author; JRS and BOO: drafted the manuscript; JM, SR, and CTC: analyzed the manuscript; BJM: served as corresponding author.

## ETHICAL APPROVAL

This case report was prepared using the appropriate ethical guidelines for human subjects.

## INFORMED CONSENT

This is a case report, and no informed consent is applicable given that no identifying information was used.

## Data Availability

The data that support the findings of this case report are available on request from the corresponding author. The data are not publicly available due to privacy or ethical restrictions.
